# Prognostic role and implications of mutation status of tumor suppressor gene ARID1A in cancer: a systematic review and meta-analysis

**DOI:** 10.18632/oncotarget.5142

**Published:** 2015-09-08

**Authors:** Claudio Luchini, Nicola Veronese, Marco Solmi, Hanbyoul Cho, Jae-Hoon Kim, Angela Chou, Anthony J. Gill, Sheila F. Faraj, Alcides Chaux, George J. Netto, Kentaro Nakayama, Satoru Kyo, Soo Young Lee, Duck-Woo Kim, George M. Yousef, Andreas Scorilas, Gregg S. Nelson, Martin Köbel, Steve E. Kalloger, David F. Schaeffer, Hai-Bo Yan, Feng Liu, Yoshihito Yokoyama, Xianyu Zhang, Da Pang, Zsuzsanna Lichner, Giuseppe Sergi, Enzo Manzato, Paola Capelli, Laura D. Wood, Aldo Scarpa, Christoph U. Correll

**Affiliations:** ^1^ Department of Pathology and Diagnostics, University and Hospital Trust of Verona, Verona, Italy; ^2^ Department of Pathology, The Johns Hopkins University, Baltimore, MD, USA; ^3^ Department of Medicine, Geriatrics Division, University of Padova, Padova, Italy; ^4^ Department of Neurosciences, University of Padova, Padova, Italy; ^5^ Department of Obstetrics and Gynecology, Gangnam Severance Hospital, Yonsei University College of Medicine, Seoul, South Korea; ^6^ Cancer Diagnosis and Pathology Group, Kolling Institute of Medical Research, St. Leonards, Australia, Sydney Vital Translational Research Centre St. Leonards Australia and University of Sydney, Sydney, NSW, Australia; ^7^ Department of Anatomical Pathology, SYDPATH St. Vincent's Hospital, Sydney, NSW, Australia; ^8^ Centro para el Desarrollo de la Investigación Científica (CEDIC), Asunción, Paraguay; ^9^ Department of Obstetrics and Gynecology, Shimane University School of Medicine, Shimane, Japan; ^10^ Lee Gil Ya Cancer and Diabetes Institute, Gachon University, Incheon, South Korea; ^11^ Department of Surgery, Seoul National University Bundang Hospital, Seongnam, South Korea; ^12^ Department of Laboratory Medicine and Keenan Research Centre, Li Ka Shing Knowledge Institute of St. Michael's Hospital, Toronto, Ontario, Canada; ^13^ Department of Biochemistry and Molecular Biology, Faculty of Biology, University of Athens, Athens, Greece; ^14^ Department of Gynecologic Oncology, Tom Baker Cancer Centre, Calgary, Alberta, Canada; ^15^ Department of Pathology and Laboratory Medicine, University of Calgary, Calgary, Alberta, Canada; ^16^ Department of Pathology and Laboratory Medicine, University of British Columbia, Vancouver, British Columbia, Canada; ^17^ Department of Systems Biology for Medicine of School of Basic Medical Sciences, and Institutes of Biomedical Sciences, Fudan University, Shanghai, China; ^18^ Department of Obstetrics and Gynecology, Hirosaki University Graduate School of Medicine, Hirosaki, Japan; ^19^ Department of Breast Surgery, Harbin Medical University Cancer Hospital, Harbin, China; ^20^ Department of Laboratory Medicine and Keenan Research Centre, Li Ka Shing Knowledge Institute of St. Michael's Hospital, Toronto, Ontario, Canada; ^21^ The Zucker Hillside Hospital, Psychiatry Research, North Shore - Long Island Jewish Health System, Glen Oaks, New York, USA; ^22^ Hofstra North Shore LIJ School of Medicine, Hempstead, New York, USA; ^23^ The Feinstein Institute for Medical Research, Manhasset, New York, USA; ^24^ Albert Einstein College of Medicine, Bronx, New York, USA

**Keywords:** ARID1A, SWI/SNF, chromatin remodeling, targeted therapy, tumor suppressor gene

## Abstract

Loss of the tumor suppressor gene AT-rich interactive domain-containing protein 1A (ARID1A) has been demonstrated in several cancers, but its prognostic role is unknown. We aimed to investigate the risk associated with loss of ARID1A (ARID1A−) for all-cause mortality, cancer-specific mortality and recurrence of disease in subjects with cancer. PubMed and SCOPUS search from database inception until 01/31/2015 without language restriction was conducted, contacting authors for unpublished data. Eligible were prospective studies reporting data on prognostic parameters in subjects with cancer, comparing participants with presence of ARID1A (ARID1A+) vs. ARID1A−, assessed either via immunohistochemistry (loss of expression) or with genetic testing (presence of mutation). Data were summarized using risk ratios (RR) for number of deaths/recurrences and hazard ratios (HR) for time-dependent risk related to ARID1A− adjusted for potential confounders. Of 136 hits, 25 studies with 5,651 participants (28 cohorts; ARID1A−: *n* = 1,701; ARID1A+: *n* = 3,950), with a mean follow-up period of 4.7 ± 1.8 years, were meta-analyzed. Compared to ARID1A+, ARID1A− significantly increased cancer-specific mortality (studies = 3; RR = 1.55, 95% confidence interval (CI) = 1.19–2.00, I^2^ = 31%). Using HRs adjusted for potential confounders, ARID1A− was associated with a greater risk of cancer-specific mortality (studies = 2; HR = 2.55, 95%CI = 1.19–5.45, I^2^ = 19%) and cancer recurrence (studies = 10; HR = 1.93, 95%CI = 1.22–3.05, I^2^ = 76%). On the basis of these results, we have demonstrated that loss of ARID1A shortened time to cancer-specific mortality, and to recurrence of cancer when adjusting for potential confounders. For its role, this gene should be considered as an important potential target for personalized medicine in cancer treatment.

## INTRODUCTION

Recent studies established that cancer development depends on both epigenetic and genomic alterations [1, 3]. Particularly, genes involved in epigenetic mechanisms establishing chromatin structure are frequently mutated in various types of human cancers [4–6]. Chromatin structure is regulated by two general classes of complexes that cooperate dynamically: the first class covalently modifies histone tails and the second remodels nucleosomes in an ATP-dependent manner. Among ATP-dependent chromatin remodelers, the so called Switch/Sucrose Non Fermentable (SWI/SNF) complexes, consisting of 9–12 subunits and possessing ATP-dependent nucleosome remodeling activity, are most commonly dysregulated in cancer [4, 6]. SWI/SNF complexes remodel nucleosome structure and can mobilize nucleosomes both by sliding and by catalyzing the ejection and insertion of histone octamers, using the energy of ATP. These complexes have important roles in gene expression regulation, even during lineage specification, and in maintaining stem cell pluripotency.

Regarding epigenetic tumor suppression function, many studies documented that SWI/SNF inactivation leads to increased sensitivity to DNA damage and suggested that these complexes have roles in the DNA damage response [7, 8]. The SWI/SNF complexes can be divided into two broad categories based upon the presence of the AT-rich interactive domain containing protein 1A-B (ARID1A/B) subunits (BAF complex) or ARID2 and Polybromo 1 (PBMR1) subunits (PBAF complex). ARID1A is an important subunit of the mammalian SWI/SNF complex (mSWI/SNF or BAF) that is mutually exclusive of the ARID1B subunit. Its expression varies during the cell cycle, being highest during G_0_-G_1_ and lowest in S and G_2_-M phases [9]. As a subunit of SWI/SNF complexes, ARID1A is thought to contribute to specific recruitment of its chromatin remodeling activity by binding transcription factors and transcriptional coactivator/corepressor complexes [10].

Several studies have related ARID1A to transcriptional regulation, particularly nuclear hormone-induced transcription and expression of cell-cycle regulators; mutations of ARID1A are frequently seen in hormone-responsive cancers, like breast and ovary cancers [5, 8, 11, 12]. Tumor suppressor genes are defined as “caretakers” if they maintain the integrity of the genome, and “gatekeepers” if they control cellular proliferation, regulating cell-cycle or promoting apoptosis. There is evidence that ARID1A has both these functions and that inactivating the tumor suppressor gene through somatic mutations and other epigenetic mechanism results in promoting tumorigenesis [13].

Although ARID1A has been established as a tumor suppressor gene through the discovery of recurrent inactivating ARID1A mutations in a broad spectrum of cancers, its prognostic role is still debated. Therefore, we aimed to investigate the prognostic role of loss of ARID1A (ARID1A−) in people with cancer regarding overall mortality, cancer-specific mortality, and recurrence of disease, hypothesizing that ARID1A− would be associated with a poorer prognosis compared to the presence of ARID1A (ARID1A+).

## RESULTS

### Search results

Altogether, the search yielded 125 non-duplicated articles. After excluding 94 articles based on title/abstract review, 31 articles were retrieved for full text review. Finally, 25 studies including 28 cohorts were included in this meta-analysis (Supplementary Figure 1).

### Study and patient characteristics

The 28 meta-analyzed cohorts followed 5,651 participants, divided in 1,701 ARID1A− and 3,950 ARID1A+ patients, for a mean period of 4.7 ± 1.8 years (range: 2–6.9 years) (Supplementary Table 1) [14–38]. The median NOS score was 7, with only one study at possible high risk of bias (Supplementary Table 2) [33].

The studies were conducted mostly in Asia (17 studies, 60.7%) [14, 18–21, 24, 26–28, 30, 31, 33, 35–38] followed by 10 studies (35.7%) [16, 17, 22, 23, 25, 29, 32, 34] in North America, and 1 study (3.6%) [15] in Australia, without any studies conducted in Europe. All studies were published after 2010. Thirteen studies (46.4%) [14, 16, 18, 19, 23–25, 28, 35–38] were conducted about gynecological cancers, 12 studies (42.3%) [15, 20, 21, 26, 29–34] about gastrointestinal cancers, and three (11.3%) [17, 22, 27] about urological cancers. Most studies (*N* = 25, 89.3%) assessed the presence of ARID1A with immunohistochemistry (tissue microarray or whole-section immunohistochemistry), while 3 (10.7%) assessed the genetic status directly (Supplementary Table 1).

Participants with ARID1A− and ARID1A+ averaged 62.1 ± 12.6 years and 58.8 ± 11.2 years (*p* = 0.40), and 54.9% and 52.7% were females (*p* = 0.41) (Supplementary Table 1). Participants with ARID1A− and ARID1A+, had a low stage of cancer according to FIGO (International Federation of Gynecology and Obstetrics) in 66.1% and 87.0% of cases (*p* = 0.26), with the corresponding figures being 53.1% and 63.6% (*p* = 0.002) using the TNM classification. Finally, 47.0% and 51.3% of participants with ARID1A− and ARID1A+ had a low grade cancer (*p* = 0.14) (Supplementary Table 1).

### Risk ratios of all-cause mortality, cancer mortality and recurrence

Pooling data from 26 studies [14–25, 27, 28, 30–38], 577 (35.3%) of 1,633 participants with ARID1A− died vs. 1,204 (32.2%) of 3,735 with ARID1A+, resulting in a non-significant group difference (RR = 1.03, 95%CI: 0.90–1.17, *p* = 0.69, I^2^ = 87%) (Table 1; Supplementary Figure 2). Similarly, across 7 studies [14, 18, 19, 22, 27, 28, 35], recurrence of cancer did not differ between the two groups, with 98/454 (21.6%) recurrences in those with ARID1A− vs. 76/373 (20.4%) in those with ARID1A+ (RR = 1.11, 95%CI: 0.98–1.25, *p* = 0.10, I^2^ = 51%) (Table 1; Supplementary Figure 3). Conversely, loss of ARID1A was associated with an increased risk of death due to cancer in three studies [16, 17, 27] compared to presence of ARID1A (138/303 = 45.5% vs. 25/130 = 19.2%; RR = 1.55, 95%CI: 1.19–2.00, *p* = 0.001, I^2^ = 31%) as shown in Table 1 and in Supplementary Figure 4.

**Table 1:** Pooled Risk Ratio Estimates For Overall Survival, Death Due To Cancer And Recurrence According To ARID1A Status

Parameter	N Studies	N of Events in loss of ARID1A group	N loss of ARID1A group	N of Events in presence of ARID1A group	N presence of ARID1A group	Risk Ratio(95% CI)	*P*-Value	Heterogeneity
All-cause mortality	26	577	1,633	1,204	3,735	1.03[0.90, 1.17]	0.69	Tau^2^ = 0.08; Chi^2^ = 191.72, df = 25 (*P* < 0.0001); I^2^ = 87%
Death due to cancer	3	138	303	25	130	**1.55[1.19, 2.00]**	**0.001**	Tau^2^ = 0.02; Chi^2^ = 2.92, df = 2 (*P* = 0.23); I^2^ = 31%
Recurrence	7	98	454	76	373	1.11[0.98, 1.25]	0.10	Tau^2^ = 0.01; Chi^2^ = 12.49, df = 6 (*P* = 0.05); I^2^ = 51%

Bolded RR values: *p* < 0.05

### Adjusted hazard ratios for all-cause mortality, cancer mortality and recurrence of disease

In secondary analyses, we investigated whether using hazard ratios (adjusted for the maximum number of the covariates available in each study) instead of risk ratios influenced the results. Altogether, the number of adjustments ranged from 0 to 10, with a mean of 3 ± 3 covariates used in the survival analyses (Supplementary Table 1).

**Figure 1:**
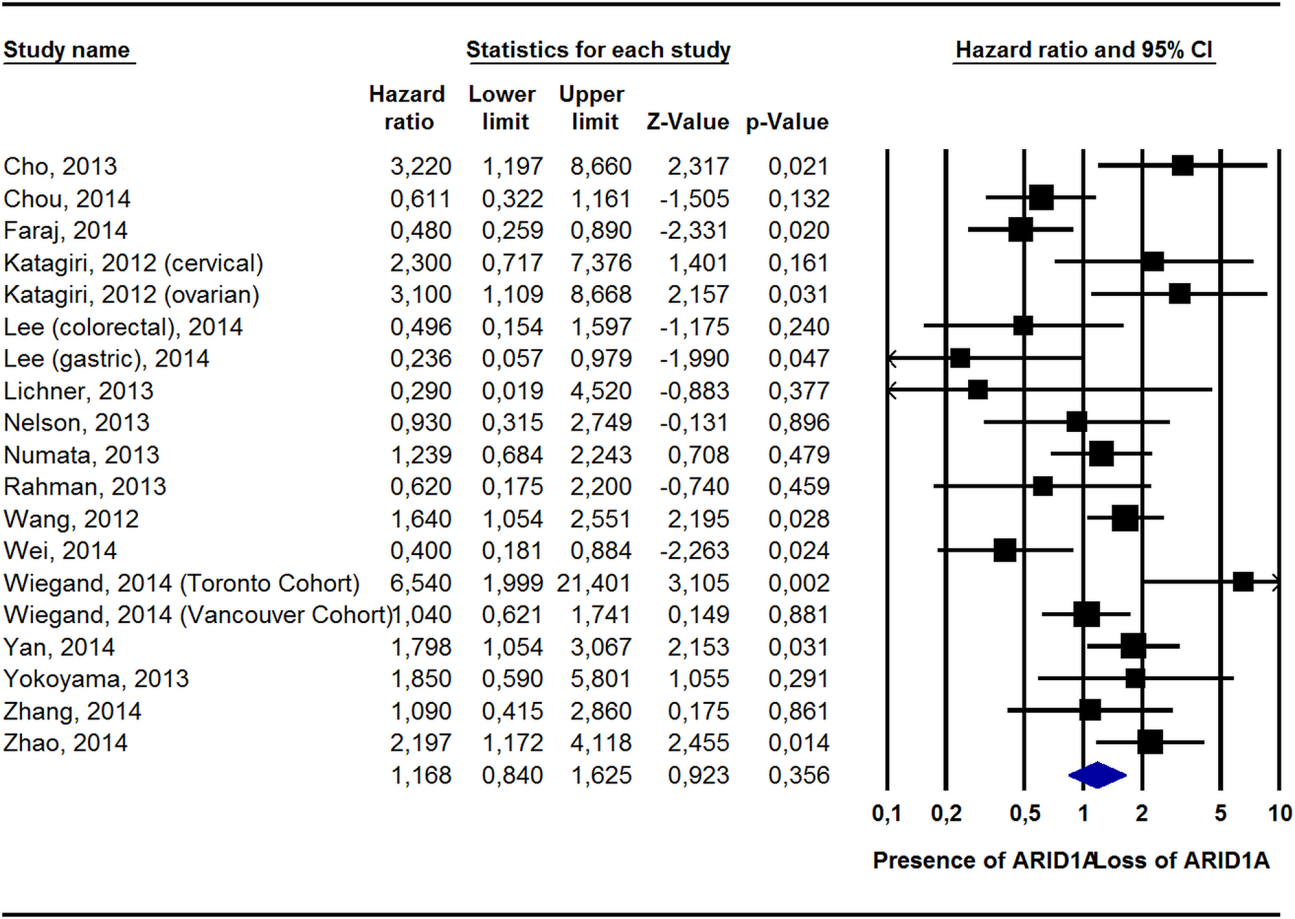
Pooled Hazard Ratio (Adjusted For Potential Confounders) For All-Cause Mortality According To ARID1A Status

**Figure 2:**
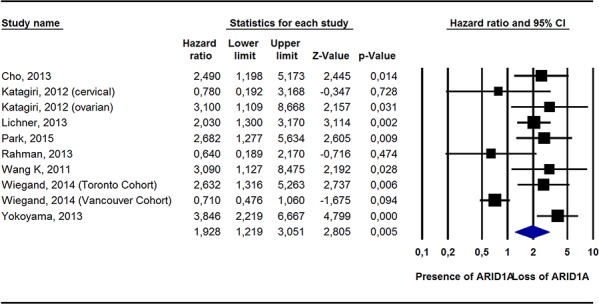
Pooled Hazard Ratio (Adjusted For Potential Confounders) For Recurrence According To ARID1A Status

Pooling data from 19 studies [14, 15, 17, 19, 21, 22, 25, 27, 28, 30, 33, 35, 37, 38], ARID1A− was still not associated with a greater risk of all-cause mortality (HR = 1.17, 95%CI: 0.84–1.63, *p* = 0.36, I^2^ = 67%). Conversely, in adjusted survival analyses, compared to ARID1A+, ARID1A− was associated with a significantly greater risk of dying from cancer (2 studies) [17, 27]; HR = 2.55, 95%CI: 1.19–2.41, *p* = 0.02, I^2^ = 19%) and of experiencing a recurrence of cancer (10 studies) [14, 18, 19, 22, 27–29, 32, 35]; HR = 1.93, 95%CI: 1.22–3.05, *p* = 0.005, I^2^ = 76%) (Table 2; Figures 1, 2; Supplementary Figure 5).

**Table 2:** Pooled Risk Ratio Estimates For Adjusted Hazard Ratios For Overall Survival, Death Due To Cancer And Recurrence According To ARID1A Status

Parameter	N Studies	Hazard Ratios(95% CI)	*P*-Value	Heterogeneity
All-cause mortality	19	1.17[0.84, 1.63]	0.36	Tau^2^ = 0.32; Chi^2^ = 54.31, df = 18 (*P* < 0.0001); I^2^ = 67%
Death due to cancer	2	**2.55 [1.19-5.45]**	**0.02**	Tau^2^ = 0.06; Chi^2^ = 1.25, df = 1 (*P* = 0.26); I^2^ = 19%
Recurrence	10	**1.93 [1.22-3.05]**	**0.005**	Tau^2^ = 0.38; Chi^2^ = 37.55, df = 9 (*P* < 0.0001); I^2^ = 76%

Bolded RR values: *p* < 0.05

### Meta-regression and sensitivity analyses

Univariable meta-regression analyses yielded only very few significant moderators for outcomes with high heterogeneity and sufficient number of studies (i.e., all-cause mortality and recurrence of cancer and corresponding analyses with adjusted HRs) (Supplementary Table 3). For all-cause mortality and cancer recurrence, only differences in percentage of low-grade cancers between ARID1A− vs. ARID1A+, country, and number of adjustments were significant moderators, while for adjusted hazard ratios no significant moderators were evident (Supplementary Table 3).

In multivariable meta-regression analyses, significant moderators emerged only for all-cause mortality, including differences in percentage of low-grade cancers between ARID1A− vs. ARID1A+ and studies about gastrointestinal and gynecological cancers compared to urological cancers (Supplementary Table 4).

Among the moderators considered for stratification (i.e., body system, country, and study quality), no significant moderators emerged explaining the absence of an association between ARID1A− and all-cause mortality (even when considering adjusted HRs), while loss of ARID1A was significantly associated with the number of recurrences of urological cancers (RR = 1.15, 95%CI: 1.07–1.23, *p* < 0.0001, I^2^ = 0%) and studies conducted in non-Asian countries (RR = 1.16, 95%CI: 1.04–1.28, *p* = 0.005) (Supplementary Table 5). Considering adjusted HRs instead of number of cancer recurrences, studies of urological cancers (HR = 2.25, 95%CI: 1.60–3.15, *p* < 0.0001, I^2^ = 78%) and those conducted in Asia (HR = 2.37, 95%CI: 1.54–3.66, *p* < 0.0001, I^2^ = 88%) significantly moderated the association between loss of ARID1A and risk of cancer recurrence adjusting survival analyses for potential confounders (Supplementary Table 5). Quality of studies was not significantly related to any of the analyzed outcomes.

### Publication bias

Funnel plots inspection indicated that publication bias was unlikely.

## DISCUSSION

To our knowledge, this is the first meta-analysis investigating the relationship between loss of ARID1A and prognosis or outcome in patients with cancer. We examined 25 prospective, observational studies involving 28 cohorts with 5,651 participants (ARID1A−: *n* = 1,701; ARID1A+: *n* = 3,950) during a mean period of 4.7 ± 1.8 years. As hypothesized, since ARID1A has been described as a potential tumor suppressor gene, loss of ARID1A was associated with increased cancer-specific mortality as well as recurrence of cancer when adjusting for potential confounders in survival analyses, while no effect was evident for all-cause mortality. Due to the high heterogeneity in four of the six meta-analyzed outcomes, a series of meta-regression and sensitivity analyses was conducted. Only all-cause mortality seemed to be partially affected by differences in low-grade tumors between ARID1A+ and ARID1A− as well as by different cancer types, i.e., gastrointestinal/gynecological vs. urological cancers. When stratifying for some potential moderators, loss of ARID1A increased the risk of cancer recurrence for urological cancers, while study origin in Asia yielded conflicting results. Notably, however, the pre-analytic and analytic variability of immunohistochemical analysis techniques is well-known, which could explain the conflicting results of some studies and the heterogeneity of the results. Thus, further studies are needed to address this point also for IHC, ideally developing a well-standardized analytic system.

Assessing the ARID1A mutational status in different types of cancers allows for a more differentiated risk evaluating. The discordance between cancer-specific mortality and time to recurrence on the one hand and all-cause mortality on the other may potentially be due to the fact that people with cancer often have many other co-morbidities and die of causes that are not directly linked to the ARID1A expression status. Furthermore, we speculate that loss of ARID1A could be involved in the progression of cancer or be correlated with locally invasive growth, mechanisms that could have less importance when a cancer is in a late stage. The expression of ARID1A may also be associated with different cancer stages [17–22]. For example, Wei et al. reported that ARID1A− was significantly associated with all-cause mortality only in stage IV patients [31]. Furthermore, Yokoyama et al. reported no differences regarding overall mortality between ARID1A+ and ARID1A− patients; yet, they described a significant association between disease free survival and ARID1A status, but only in TNM stage III-IV cancer patients [35]. Notably, these authors described a significant association between loss of ARID1A and chemoresistance suggesting that ARID1A− may be a factor more important for predicting the risk of recurrence, affecting more the disease free survival than overall survival [35]. In order to determine whether the expression level of ARID1A has any effect on overall survival, further prospective studies are needed, ideally differentiated by stage.

Loss of ARID1A function is associated with dysregulation of the PI3K/Akt signaling pathway, which may have a synergistic effect on tumor development [39, 40]. Moreover, an inverse relationship has been documented between ARID1A and TP53 in uterine endometrioid [40, 41], gastric [29] and esophageal carcinomas [42], and between ARID1A and Phosphatase and TENsin homologue (PTEN) in colorectal and serous ovarian carcinomas [7]. Furthermore, a very important issue in the prognostic analysis associated with ARID1A mutational status can be related to defects in mismatch repair, of which microsatellite instability (MSI) is the phenotype. MSI involves short repeats of mono- or oligonucleotides that are typically also present in ARID1A, and MSI is associated with a remarkably high rate of sequence mutation in cancer cells [13]. ARID1A mutation has been associated with MSI in gastrointestinal cancers [15, 29]. Lastly, ARID1A− has been reported to be correlated with Epstein-Barr virus (EBV)-associated gastric cancers [43]. Wang et al. found ARID1A mutations in 47% of EBV-infected, microsatellite stable gastric tumor samples, which was significantly higher than in microsatellite stable gastric tumors without EBV-infection (percentage not indicated in the paper) [29]. MSI and EBV-infection are associated with better prognosis and are also associated with ARID1A loss. Considering both MSI and EBV infection, ARID1A loss was correlated with poor prognosis only in gastric cancers without MSI and EBV infection [43]. Thus, due to this complex interplay between partially offsetting interactions, it will be important for better understanding the prognostic role of ARID1A to identify the stages and subgroups in different types of cancers that can be affected by ARID1A mutational status.

From the targeted therapy's point of view, interestingly, a recent systematic review of genetic vulnerability across cancer cell lines identified ARID1B, the ARID1A mutually exclusive subunit in mSWI/SNF (BAF) complex, as the top gene required for cancer cell survival with inactivating ARID1A mutations [44]. ARID1B was required for the stable assembly of BAF complex in ARID1A− cells. Silencing ARID1B impaired cellular proliferation in cancer cells with ARID1A mutations, but not in cells with wild-type ARID1A, suggesting that ARID1B is a potential therapeutic target for cancers with ARID1A mutation. The involvement of ARID1A in maintaining genomic stability makes cancers with ARID1A mutations potential candidates for therapeutic approaches based on synthetic lethality: an ARID1A− deficient tumor, with its intrinsic genomic instability, may be vulnerable to therapies targeting molecular pathways involving genome maintenance. Due to the possible correlations between ARID1A mutations and other pathways (e.g. PI3K pathway), it will be interesting to investigate the effect of inhibitors of other pathways on tumors with different ARID1A mutational status. With more emerging epigenetic cancer therapies, it will be important to better understand the landscape of ARID1A− containing mSWI/SNF targets and the epigenetic alterations that follow ARID1A mutations. The goal is the application of personalized medicine to ARID1A deficient tumors and perhaps even to precancerous lesions, generating a next-generation histopathologic diagnosis [45, 46]. Despite these very heterogeneous genetic and epigenetic interactions of ARID1A with other important molecular mechanisms, our statistically significant results of the prognostic value of ARID1A mutation on cancer recurrence and death due to cancer have relevant research implications.

The findings of our meta-analysis, however, should be interpreted within its limitations, the most important of which is represented by the heterogeneity of the results. It is likely due to the fact that the included studies had different baseline characteristics (in particular regarding tumor grading and stage) that could affect the results. Taking as example all-cause mortality, differences in low-grade cancers partly explained the heterogeneity of our findings. Therefore, future studies with more homogeneity of tumor grading and stage are needed, at least in subgroup analyses. Furthermore, we were not able to control our analyses for some factors that are independently associated with overall or cancer-specific mortality or with cancer recurrence, including smoking, number/type of medical morbidities and medications, and obesity, because they are not analysed in the selected papers. Lastly, about one half of the included studies did not consider other genes, although these are likely important too since ARID1A is involved in several pathways. Therefore, in the future the ability to adjust for the mutations of other genes could be fundamental for a better interpretation of the role of ARID1A and cancer outcomes.

In conclusion, loss of ARID1A shortened time to cancer-specific mortality as well as to recurrence of disease when adjusting for potential confounders. Since many types of neoplasms are characterized by loss of ARID1A, further studies are needed to find ways to leverage ARID1A findings for developing targeted therapeutic interventions and understand more in depth the heterogeneity of the results.

## MATERIALS AND METHODS

### Data sources and literature search strategy

Two investigators (C.L., N.V.) independently conducted a literature search using PubMed and SCOPUS without language restriction, from database inception until 01/31/2015, for prospective studies comparing all-cause mortality, cancer mortality and recurrence of cancer in patients with a diagnosis of cancer with loss vs. presence of expression of ARID1A. In PubMed and SCOPUS, controlled vocabulary terms and the following keywords were used: (“ARID1A” OR “AT-rich interactive domain-containing protein 1A” OR “BAF250a”) AND (“cancer” OR “neoplasm” OR “carcinoma”) AND (“Mortality” OR “Mortalities” OR “Case Fatality Rate” OR “Case Fatality Rates” OR “Death Rate” OR “Death Rates” OR “survival” OR “prognosis” OR “hazard ratio” OR HR OR “relative risk” OR RR). Conference abstracts and reference lists of included articles and those relevant to the topic were hand-searched for identification of additional, potentially relevant articles. Any inconsistencies were resolved by consensus.

### Study selection

Inclusion criteria for this meta-analysis were: 1) prospective, observational cohort study, 2) immunohistochemical or genetic investigation of ARID1A, 3) diagnosis of cancer, 4) data about mortality or cancer recurrence. Since most mutations in ARID1A are insertions or deletions, resulting in a truncated protein that is prone to rapid degradation, ARID1A gene mutations are highly associated with loss of protein expression. For this reason, we included also the studies based on immunohistochemical analysis, which can be used as a surrogate marker for the underlying gene mutation.

Exclusion criteria were: 1) no presence of cancer, 2) no data about relevant outcomes in the title/abstract, 3) did not compare patients with ARID1A− vs ARID1A+, and 4) *in vitro* or animal studies.

### Data extraction

Two investigators (N.V. and M.S.) extracted key data from the included articles and a third independent investigator (C.C.) checked these data. For each article, we extracted data about authors, year of publication, country, type of cancer, exclusion criteria, other genes or proteins analyzed, participant characteristics according to ARID1A status (e.g., age, percentage of females, tumor stage and grading), methods of ARID1A assessment, number of adjustments in survival analysis, and duration of follow-up (Supplementary Table 1). When some information about ARID1A or outcomes was missing, first and/or corresponding authors of the original article were contacted at least four times to obtain unpublished data.

### Outcomes

The primary outcomes were number of deaths independent of the cause (all-cause mortality), number of deaths due to cancer, and number of cancer recurrences after treatment (e.g., chemotherapy, surgery, radiotherapy) during follow-up period depending on the loss or the presence of ARID1A.

### Assessment of study quality

We used the Newcastle-Ottawa Scale (NOS) (http://www.ohri.ca/programs/clinical_epidemiology/oxford.htm) to evaluate study quality, with a score of ≤ 5 (out of 9) indicating high risk of bias (Supplementary Table 2) [47]. This systematic review was conducted following the Meta-Analysis Of Observational Studies in Epidemiology (MOOSE) guidelines and Preferred Reporting Items for Systematic reviews and Meta-Analyses (PRISMA) statement [48, 49].

### Data synthesis and statistical analysis

Analyses were performed using Comprehensive Meta-Analysis (CMA) 3 (http://www.meta-analysis.com).

In primary analyses, pooled risk ratios (RRs) and 95% CIs of all-cause mortality, cancer mortality and recurrences in patients with ARID1A+ and ARID1A− tumors were calculated using DerSimonian-Laird random-effects models [50]. In secondary analyses, pooled, hazard ratios (HRs) with 95%CIs adjusted for the maximum number of covariates available, were also calculated for providing additional information if the relationship between ARID1A status and outcomes was influenced by potential confounders. Heterogeneity across studies was assessed by the Cochrane I^2^ metric and chi square statistics. Given significant heterogeneity (*p* < 0.05), we conducted a series of univariable and multivariable meta-regression analyses according to ARID1A status considering each of the outcomes [51].

The following moderators were tested: country (Asia vs. other continents), body system (urological, gastrointestinal, or gynecological), sample size, study quality (NOS score), number of adjustments, methods of the assessment of ARID1A, duration of follow-up, and differences between ARID1A+ and ARID1A− in age, percentage of females, tumor stage (divided in Tumor, Nodes, Metastasis (TNM) stage 1–2, indicating low stage, and TNM 3–4, indicating higher stage), tumor grading (divided in G1–2, indicating low grade, and G3–4, indicating higher grade). Any moderators with a *p*-value ≤ 0.10 in univariable meta-regression analyses for a specific ARID1A contrast group were entered into a backward elimination multivariable meta-regression analysis for that ARID1A contrast group.

We also conducted stratified analyses exploring effects of the following pre-specified moderators: body system (gastrointestinal, gynecological or urological), study origin (Asia vs. other continents), and study quality (median split of the NOS score [NOS = 7]). All final inferential statistics used alpha = 0.05.

Lastly, we assessed the presence of publication bias by visual inspection of Funnel plots.

## SUPPLEMENTARY FIGURES AND TABLES



## References

[1] Biegel JA, Busse TM, Weissman BE (2014). SWI/SNF chromatin remodeling complexes and cancer. Am J Med Genet C Semin Med Genet.

[2] Choi JD, Lee JS (2013). Interplay between epigenetics and genetics in cancer. Genomic Inform.

[3] Feinberg AP (2014). Epigenetic stochasticity, nuclear structure and cancer: the implications for medicine. J Intern Med.

[4] Wilson GW, Roberts CW (2011). SWI/SNF nucleosome remodelers and cancer. Nat Rew Cancer.

[5] Jones S, Stransky N, McCord CL, Cerami E, Lagowski J, Kelly D, Angiuoli SV, Sausen M, Kann L, Shukla M, Makar R, Wood LD, Diaz LA (2014). Genomic analyses of gynaecologic carcinosarcomas reveal frequent mutations in chromatin remodelling genes. Nat Commun.

[6] Varela I, Tarpey P, Raine K, Huang D, Ong CK, Stephens P, Davies H, Jones D, Lin ML, Teague J, Bignell G, Butler A, Cho J (2011). Exome sequencing identifies frequent mutation of the SWI/SNF complex gene PBMR1 in renal carcinoma. Nature.

[7] Kadoch C, Hargreaves DC, Hodges C, Elias L, Ho L, Ranish J, Crabtree GR (2013). Proteomic and bioinformatic analysis of mammalian SWI/SNF complexes identifies extendive roles in human malignangcy. Nat Genet.

[8] Wu JN, Roberts CW (2013). ARID1A mutations in cancer: another epigenetic tumor suppressor?. Cancer Discov.

[9] Flores-Alcantar A, Gonzalez-Sandoval A, Escalante-Alcade D, Lomeli H (2011). Dynamics of expression of ARID1A and ARID1B subunits in mouse embryos and in cells during the cell cycle. Cell Tissue Res.

[10] Nie Z, Xue Y, Yang D, Zhou S, Deroo BJ, Archer TK, Wang W (2000). A specificity and targeting subunit of a human SWI/SNF family-related chromatin-remodeling complex. Mol Cell Biol.

[11] Mao TL, Shih IeM (2013). The roles of ARID1A in gynecologic cancer. J Gynecol Oncol.

[12] Samartzis E, Noske A, Dedes KJ, Fink D, Imesch P (2013). ARID1A mutations and PI3K/AKT pathway alterations in endometriosis-associated ovarian carcinomas. Int J Mol Sci.

[13] Wu RC, Wang TL, Shih IeM (2014). The emerging roles of ARID1A in tumor suppression. Cancer Biol Ther.

[14] Cho H, Kim JS, Chung H, Perry C, Lee H, Kim JH (2013). Loss of ARID1A/BAF250a expression is linked to tumor progression and adverse prognosis in cervical cancer. Hum Pathol.

[15] Chou A, Toon CW, Clarkson A, Sioson L, Houang M, Watson N, DeSilva K, Gill AJ (2014). Loss of ARID1A expression in colorectal carcinoma is strongly associated with mismatch repair deficiency. Hum Pathol.

[16] Fadare O, Renshaw IL, Liang SX (2012). Does the Loss of ARID1A (BAF-250a) Expression in Endometrial Clear Cell Carcinomas Have Any Clinicopathologic Significance?. A Pilot Assessment. J Cancer.

[17] Faraj SF, Chaux A, Gonzalez-Roibon N, Munari E, Ellis C, Driscoll T, Schoenberg MP, Bivalacqua TJ, Shih IeM, Netto GJ (2014). ARID1A immunohistochemistry improves outcome prediction in invasive urothelial carcinoma of urinary bladder. Hum Pathol.

[18] Katagiri A, Nakayama K, Rahman MT, Rahman M, Katagiri H, Ishikawa M, Ishibashi T, Iida K, Otsuki Y, Nakayama S, Miyazaki K (2012). Frequent loss of tumor suppressor ARID1A protein expression in adenocarcinomas/adenosquamous carcinomas of the uterine cervix. Int J Gynecol Cancer.

[19] Katagiri A, Nakayama K, Rahman MT, Rahman M, Katagiri H, Nakayama N, Ishikawa M, Ishibashi T, Iida K, Kobayashi H, Otsuki Y, Nakayama S, Miyazaki K (2012). Loss of ARID1A expression is related to shorter progression-free survival and chemoresistance in ovarian clear cell carcinoma. Mod Pathol.

[20] Kim MJ, Gu MJ, Chang HK, Yu E (2015). Loss of ARID1A expression is associated with poor prognosis in small intestinal carcinoma. Histopathology.

[21] Lee SY, Kim DW, Lee HS, Ihn MH, Oh HK, Park do J (2015). Loss of AT-Rich Interactive Domain 1A Expression in Gastrointestinal Malignancies. Oncology.

[22] Lichner Z, Scorilas A, White NM, Girgis AH, Rotstein L, Wiegand KC, Latif A, Chow C, Huntsman D, Yousef GM (2013). The chromatin remodeling gene ARID1A is a new prognostic marker in clear cell renal cell carcinoma. Am J Pathol.

[23] Lowery WJ, Schildkraut JM, Akushevich L, Bentley R, Marks JR, Huntsman D, Berchuck A (2012). Loss of ARID1A− associated protein expression is a frequent event in clear cell and endometrioid ovarian cancers. Int J Gynecol Cancer.

[24] Maeda D, Mao TL, Fukayama M, Nakagawa S, Yano T, Taketani Y, Shih IeM (2010). Clinicopathological significance of loss of ARID1A immunoreactivity in ovarian clear cell carcinoma. Int J Mol Sci.

[25] Nelson GS, Pink A, Lee S, Han G, Morris D, Ogilvie T, Duggan MA, Köbel M (2013). MMR deficiency is common in high-grade endometrioid carcinomas and is associated with an unfavorable outcome. Gynecol Oncol.

[26] Numata M, Morinaga S, Watanabe T, Tamagawa H, Yamamoto N, Shiozawa M, Nakamura Y, Kameda Y, Okawa S, Rino Y, Akaike M, Masuda M, Miyagi Y (2013). The clinical significance of SWI/SNF complex in pancreatic cancer. Int J Oncol.

[27] Park JH, Lee C, Suh JH, Chae JY, Kim HW, Moon KC (2015). Decreased ARID1A expression correlates with poor prognosis of clear cell renal cell carcinoma. Hum Pathol.

[28] Rahman M, Nakayama K, Rahman MT, Katagiri H, Katagiri A, Ishibashi T, Ishikawa M, Iida K, Miyazaki K (2013). Clinicopathologic analysis of loss of AT-rich interactive domain 1A expression in endometrial cancer. Hum Pathol.

[29] Wang K, Kan J, Yuen ST, Shi ST, Chu KM, Law S, Chan TL, Kan Z, Chan AS, Tsui WY, Lee SP, Ho SL, Chan AK (2011). Exome sequencing identifies frequent mutation of ARID1A in molecular subtypes of gastric cancer. Nat Genet.

[30] Wang DD, Chen YB, Pan K, Wang W, Chen SP, Chen JG, Zhao JJ, Lv L, Pan QZ, Li YQ, Wang QJ, Huang LX, Ke ML (2012). Decreased expression of the ARID1A gene is associated with poor prognosis in primary gastric cancer. PLoS One.

[31] Wei XL, Wang DS, Xi SY, Wu WJ, Chen DL, Zeng ZL, Wang RY, Huang YX1, Jin Y, Wang F, Qiu MZ, Luo HY, Zhang DS (2014). Clinicopathologic and prognostic relevance of ARID1A protein loss in colorectal cancer. World J Gastroenterol.

[32] Wiegand KC, Sy K, Kalloger SE, Li-Chang H, Woods R, Kumar A, Streutker CJ, Hafezi-Bakhtiari S, Zhou C, Lim HJ, Huntsman DG, Clarke B, Schaeffer DF (2014). ARID1A/BAF250a as a prognostic marker for gastric carcinoma: a study of 2 cohorts. Hum Pathol.

[33] Yan HB, Wang XF, Zhang Q, Tang ZQ, Jiang YH, Fan HZ, Sun YH, Yang PY, Liu F (2014). Reduced expression of the chromatin remodeling gene ARID1A enhances gastric cancer cell migration and invasion via downregulation of E-cadherin transcription. Carcinogenesis.

[34] Ye J, Zhou Y, Weiser MR, Gönen M, Zhang L, Samdani T, Bacares R, DeLair D, Ivelja S, Vakiani E, Klimstra DS, Soslow RA, Shia J (2014). Immunohistochemical detection of ARID1A in colorectal carcinoma: loss of staining is associated with sporadic microsatellite unstable tumors with medullary histology and high TNM stage. Hum Pathol.

[35] Yokoyama Y, Matsushita Y, Shigeto T, Futagami M, Mizunuma H (2014). Decreased ARID1A expression is correlated with chemoresistance in epithelial ovarian cancer. J Gynecol Oncol.

[36] Zhang X, Zhang Y, Yang Y, Niu M, Sun S, Ji H, Ma Y, Yao G, Jiang Y, Shan M, Zhang G, Pang D (2012). Frequent low expression of chromatin remodeling gene ARID1A in breast cancer and its clinical significance. Cancer Epidemiol.

[37] Zhang ZM, Xiao S, Sun GY, Liu YP, Zhang FH, Yang HF, Li J, Qiu HB, Liu Y, Zhang C, Kang S, Shan BE (2014). The clinicopathologic significance of the loss of BAF250a (ARID1A) expression in endometrial carcinoma. Int J Gynecol Cancer.

[38] Zhao J, Liu C, Zhao Z (2014). ARID1A: a potential prognostic factor for breast cancer. Tumour Biol.

[39] Yamamoto S, Tsuda H, Takano M, Tamai S, Matsubara O (2012). Loss of ARID1A protein expression occurs as an early event in ovarian clear cell carcinoma development and frequently coexists with PIK3CA mutations. Mod Pathol.

[40] Bosse T, ter Haar NT, Seeber LM, v Diest PJ, Hes FJ, Vasen HF, Nout RA, Creutzberg CL, Morreau H, Smit VT (2013). Loss of ARID1A expression and its relationship with Pi3K-Akt pathway alterations, TP53 and microsatellite instability in endometrial cancer. Mod Pathol.

[41] Allo G, Bernardini MQ, Wu RC, Shih IeM, Kalloger S, Pollett A, Gilks CB, Clarke BA (2014). ARID1A loss correlates with mismatch repair deficiency and intact p53 expression in high-grade endometrial carcinomas. Mod Pathol.

[42] Streppel MM, Lata S, DelaBastide M, Montgomery EA, Wang JS, Canto MI, Macgregor-Das AM, Pai S, Morsink FH, Offerhaus GJ, Antoniou E, Maitra A, McCombie WR (2014). Next-generation sequencing of endoscopic biopsies identifies ARID1A as a tumor-suppressor gene in Barrett's esophagus. Oncogene.

[43] Abe H, Maeda D, Hino R, Otake Y, Isogai M, Ushiku AS, Matsusaka K, Kunita A, Ushiku T, Uozaki H, Tateishi Y, Hishima T, Iwasaki Y (2012). ARID1A expression loss in gastric cancer: pathway-dependent roles with and without Epstein-Barr virus infection and microsatellite instability. Virchows Arch.

[44] Helming KC, Wang X, Wilson BG, Vazquez F, Haswell JR, Manchester HE, Kim Y, Kryukov GV, Ghandi M, Aguirre AJ, Jagani Z, Wang Z, Garraway LA (2014). ARID1B is a specific vulnerability in ARID1A− mutant cancers. Nat Med.

[45] Luchini C, Capelli P, Fassan M, Simbolo M, Mafficini A, Pedica F, Ruzzenente A, Guglielmi A, Corbo V, Scarpa A (2014). Next generation histopathologic diagnosis: a lesson from a hepatic carcinosarcoma. J Clin Oncol.

[46] Fassan M, Simbolo M, Bria E, Mafficini A, Pilotto S, Capelli P, Bencivenga M, Pecori S, Luchini C, Neves D, Turri G, Vicentini C, Montagna L (2014). High-throughput mutation profiling identifies novel molecular dysregulation in high-grade intraepithelial neoplasia and early gastric cancers. Gastric Cancer.

[47] Wells GA, Shea B, O'Connell D, Peterson J, Welch V, Losos M, Tugwell P The Newcastle-Ottawa Scale (NOS) for assessing the quality of nonrandomised studies in metaanalyses. http://www.ohri.ca/programs/clinical_epidemiology/oxford.htm.

[48] Stroup DF, Berlin JA, Morton SC, Olkin I, Williamson GD, Rennie D, Moher D, Becker BJ, Sipe TA, Thacker SB (2000). Meta-analysis of observational studies in epidemiology: a proposal for reporting. Meta-analysis Of Observational Studies in Epidemiology (MOOSE) group. JAMA.

[49] Liberati A, Altman DG, Tetzlaff J, Mulrow C, Gøtzsche PC, Ioannidis JP, Clarke M, Devereaux PJ, Kleijnen J, Moher D (2009). The PRISMA statement for reporting systematic reviews and meta-analyses of studies that evaluate healthcare interventions: explanation and elaboration. BMJ.

[50] DerSimonian R, Laird N (1986). Meta-analysis in clinical trials. Control Clin Trials.

[51] Higgins JP, Thompson SG (2002). Quantifying heterogeneity in a meta-analysis. Stat Med.

