# Supplementary material for: Prognostic role and implications of mutation status of tumor suppressor gene ARID1A in cancer: a systematic review and meta-analysis
Source: Oncotarget. 2015 Sep 8;6(36):39088–97. doi: 10.18632/oncotarget.5142 (PMC4770758; doi:10.18632/oncotarget.5142)
Supplement: Supplementary file 1 [file oncotarget-06-39088-s001.pdf]

SUPPLEMENTARY FIGURES AND TABLES

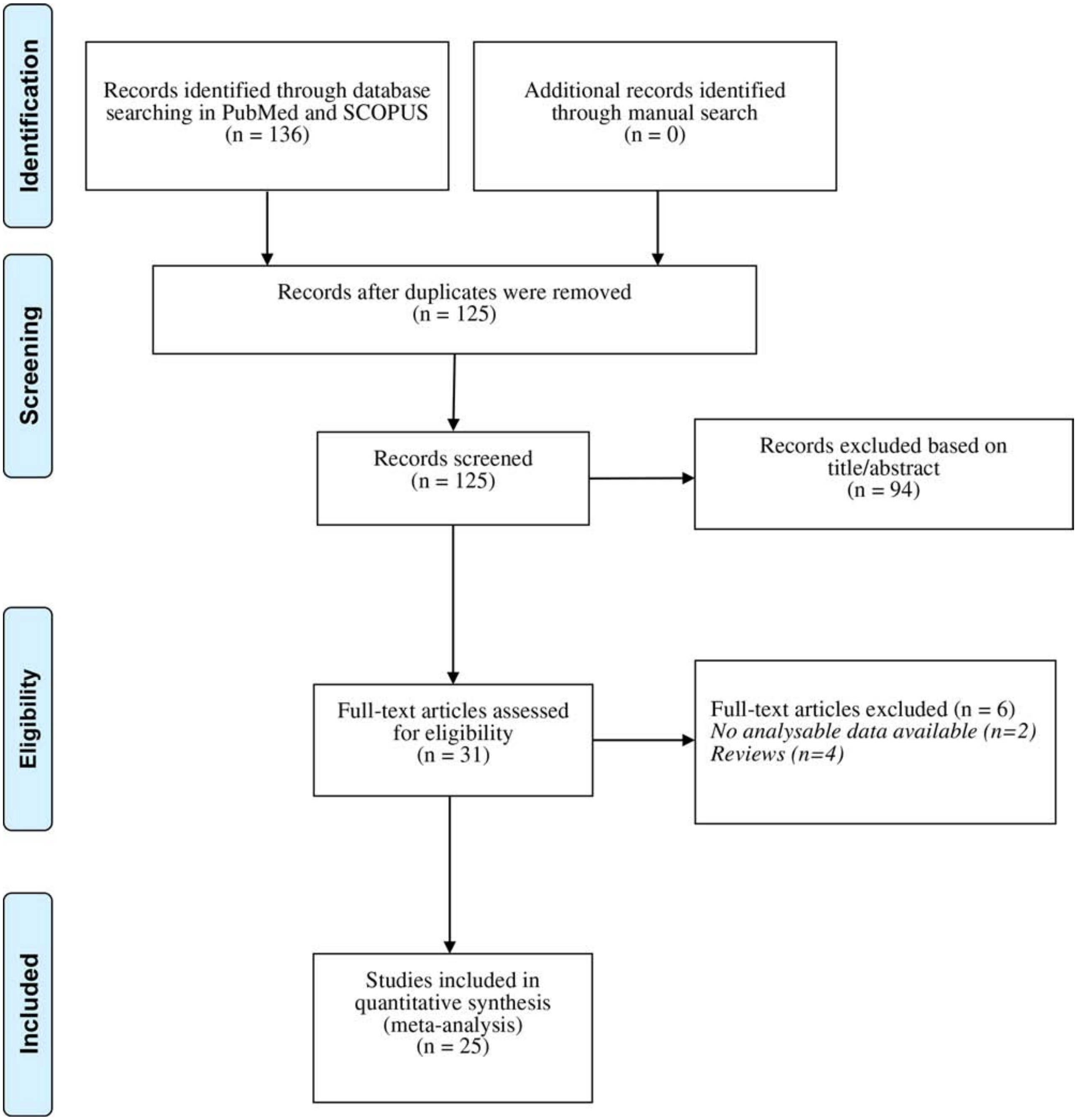

Supplementary Figure S1: PRISMA Flow-Diagram.

Study name	Risk ratio	Lower limit	Upper limit	Z-Value	p-Value
	Statistics for each study				
Cho, 2013	1,249	0,993	1,570	1,902	0,057
Chou, 2014	0,886	0,797	0,984	-2,262	0,024
Fadare, 2012	2,344	0,795	6,908	1,544	0,122
Faraj, 2014	0,613	0,413	0,908	-2,439	0,015
Katagiri (cervical), 2012	1,054	0,827	1,344	0,423	0,673
Katagiri (ovarian), 2012	1,376	0,753	2,518	1,037	0,300
Kim, 2014	2,000	1,329	3,009	3,325	0,001
Lee (colorectal), 2014	0,703	0,493	1,002	-1,951	0,051
Lee (gastric), 2014	0,856	0,733	0,998	-1,981	0,048
Lichner, 2013	0,479	0,406	0,565	-8,736	0,000
Lowery (clear cells), 2013	1,079	0,775	1,504	0,451	0,652
Lowery (endometroid), 2013	0,872	0,694	1,096	-1,172	0,241
Maeda, 2010	1,016	0,855	1,206	0,178	0,859
Nelson, 2013	0,731	0,451	1,184	-1,274	0,203
Park, 2015	1,340	1,147	1,565	3,698	0,000
Rahman, 2013	0,964	0,822	1,131	-0,447	0,655
Wang, 2012	1,959	1,428	2,689	4,165	0,000
Wei (stage IV colorectal cancer), 2014	0,585	0,329	1,042	-1,820	0,069
Wiengand (Toronto Cohort), 2014	1,875	0,977	3,600	1,889	0,059
Wiengand (Vancouver Cohort), 2014	0,032	0,002	0,599	-2,303	0,021
Yan, 2014	1,735	1,225	2,457	3,105	0,002
Ye, 2014	1,173	0,876	1,572	1,071	0,284
Yokoyama, 2013	0,489	0,299	0,800	-2,850	0,004
Zhang, 2012	1,140	0,919	1,414	1,189	0,235
Zhang, 2014	1,444	0,980	2,130	1,857	0,063
Zhao, 2014	1,118	1,035	1,209	2,812	0,005
	1,027	0,902	1,170	0,404	0,686

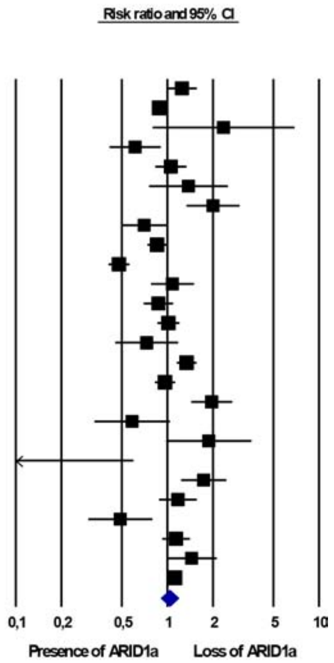

Supplementary Figure S2: Pooled Risk Ratios For All-Cause Mortality According To ARID1A Status.

Study name	Risk ratio	Lower limit	Upper limit	Z-Value	p-Value
	Statistics for each study				
Cho, 2013	1,392	1,007	1,924	2,005	0,045
Katagiri, 2012 (cervical)	0,974	0,705	1,346	-0,158	0,875
Katagiri, 2012 (ovarian)	3,176	0,924	10,922	1,834	0,067
Lichner, 2013	1,155	1,043	1,279	2,778	0,005
Park, 2015	1,123	1,014	1,243	2,231	0,026
Rahman, 2013	0,901	0,743	1,092	-1,065	0,287
Yokoyama, 2014	8,333	0,533	130,377	1,511	0,131
	1,109	0,981	1,253	1,646	0,100

Risk ratio and 95% CI

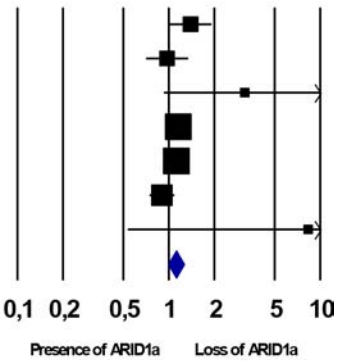

Supplementary Figure S3: Pooled Risk Ratios For Recurrence According To ARID1A Status.

Study name	Statistics for each study				
	Risk ratio	Lower limit	Upper limit	Z-Value	p-Value
Fadare, 2012	2,344	0,795	6,908	1,544	0,122
Faraj, 2014	1,930	1,251	2,976	2,975	0,003
Park, 2015	1,383	1,221	1,567	5,092	0,000
	1,546	1,194	2,002	3,304	0,001

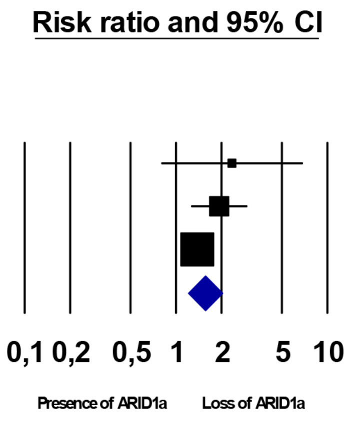

Supplementary Figure S4: Pooled Risk Ratios For Death Due To Cancer According To ARID1A Status.

Study name	Hazard ratio	Lower limit	Upper limit	Z-Value	p-Value
	Statistics for each study				
Faraj, 2014	3,810	1,424	10,196	2,663	0,008
Park, 2015	1,753	0,684	4,493	1,169	0,242
	2,549	1,192	5,451	2,412	0,016

Hazard ratio and 95% CI

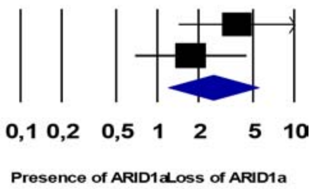

Supplementary Figure S5: Pooled Hazard Ratio (Adjusted For Potential Confounders) For Death Due To Cancer According To ARID1A Status.

Supplementary Table S1: Characteristics Of The Studies According To The Expression Of ARID1A

				ARID1A−					ARID1A+							
Study Author, Year (Country)	Type of cancer	Exclusion criteria	Other genes/ proteins abnor-malities	Number of partici-pants	N. of females (%)	Mean Age ± SD	TNM stage at baseline (number, %)	Tumor Grading	Number of partici-pants	N. of females (%)	Mean Mean Age ± SD	TNM stage at baseline (number, %)	Tumor Grading	Methods of ARID1A analysis (if IHC: clone specified) #	Number of adjust-ments	Mean Follow-up period (years)
**Cho, 2013 (Korea)**	Cervical cancer	No data at follow-up; no clinicopatho-logical data	No	27	100%	49.0 ± 14.3	FIGO I-II 81.5% FIGO IV: 18.5%	G1-G2: 48.2% G3: 51.8%	120	100%	48.5± 11.1	FIGO I-II: 96.6% FIGO IV: 3.4%	G1-G2: 66.9% G3: 33.1%	TMA-IHC (1)	4	4.5
**Chou, 2014 (Australia)**	Colorectal cancer	NA	BRAF (V600E). MMR status	74	62.2%	77.0 ± 8.0	Stage: I-II: 56.7% III-IV: 43.3%	G1-G2: 42.9% G3: 57.1%	1334	50.9%	71.2± 12.6	Stage I-II: 53.3% III-IV: 46.7%	G1-G2: 82.0% G3: 18.0%	TMA-IHC (2)	6	5.3
**Fadare, 2012 (USA)**	Endometrial clear cell carcinoma	Not pure histology (considered only pure clear cell cercinomas)	No	5	100%	63 (range 61-78)	FIGO I-II: 0% III-IV: 100%	NA	17	100%	69 (range 54-87)	FIGO I-II: 94.1% III-IV: 5.9%	NA	Whole-section IHC (1)	0	3.9
**Faraj, 2014 (USA)**	Invasive urothelial carcinoma of urinary bladder	TMA with normal urothelium	No	85	8.2%	65.3± 11.3	Stage 0-I-II: 43.5% III-IV: 56.5%	NA	37	45.9%	67.1± 9.8	Stage I-II: 70.3% III-IV: 29.7%	NA	TMA-IHC (2)	5	3.9
**Katagiri, 2012 (Japan) (cervical)**	Adeno-(squamous) carcinomas of uterine cervix	Squamous carcinomas (focus on adenocar-cinomas)	Ki-67	14	100%	49.8± 13.5	Stage I-II: 78.6% III-IV: 21.4%	NA	31	100%	46.8± 10.9	Stage I-II: 74.2% III-IV: 25.8%	NA	TMA- IHC (1)	0	3.8
**Katagiri, 2012 (Japan)**	Ovarian clear cell carcinoma	NA	Ki-67	9	100%	53.1± 3.3	Stage I-II: 44.4% III-IV: 55.6%	G3: 100%	51	100%	53.2± 9.1	StageI-II: 80.4%III-IV: 19.6%	G3: 100%	TMA-IHC (1)	3	3.8

(*Continued*)

				ARID1A−					ARID1A+							
**Kim, 2014 (Korea)**	Small intestinal carcinoma	Cases with core loss in IHC slides	p53	96	38.9%	NA	NA	G1-G2: 63.9% G3-G4: 36.1%	82	37.3%	NA	NA	G1-G2: 79.6% G3-G4: 20.4%	TMA-IHC (2)	0	4.6
**Lee, 2014 (Korea)**	Colorectal cancer	Hereditary Syndromes	No	12	41.7%	65.3 ± 11.8	Stage I-II: 16.7%. III-IV: 83.3%	G1-G2:. 91.7% G3: 8.3%	184	45.7%	59.4 ± 12.1	Stage I-II: 12.5%. III-IV: 87.5%	G1-G2:. 86.9% G3: 13.1%	TMA-IHC (2)	5	5
**Lee, 2014 (Korea)**	Gastric cancer	Hereditary Syndromes	No	22	40.9%	63.4 ± 9.9	Stage I-II: 68.2%. III-IV: 31.8%	NA	248	36.4%	59.5± 11.5	Stage I-II: 63.6%. III-IV:. 36.4%	NA	TMA-IHC (2)	4	5
**Lichner, 2013 (Canada)**	Renal cancer	NA	No	139	43.1%	60.1± 1.9	Stage I: 47.9%; II-IV: 52.1%	G1: 57.9%; G2-G3: 42.1%	265	56.9%	59.6 ± 1.4	Stage I: 56.9%; II-IV: 43.1%	G1: 42.1%; G2-G3: 57.9%	TMA-ICH (3)	2	5
**Lowery, 2012 (USA)**	Clear cell and endo-metrioid ovarian cancer	No	No	96	100%	>49: 61.5%	Stage I-II: 64.2%. III-IV: 35.8%	G1-G2: 46.8% G3: 53.2%	116	100%	>49: 70.7%	Stage I-II: 58.3% III-IV: 41.7%	G1-G2: 36.0% G3: 64.0%	Whole-section IHC (3)	0 for both cancers	5.9
**Maeda, 2010 (Japan)**	Ovarian clear cell carcinoma	No data at follow-up; no clinicopatho-logical data	No	83	100%	>49: 69.0%	Stage I-II: 74.3% III/IV: 25.7%	Nuclear atypia: mild-moderate 91.4% Severe 8.6%	52	100%	>49: 58.1%	Stage I-II: 74.5% III/IV: 25.5%	Nuclear atypia: mild-moderate 83.9% Severe 16.1%	TMA-IHC (4)	0	5.0
**Nelson, 2013 (Canada)**	Endometrial carcinoma	No	MMR genes	19	100%	64.3± 8.6	Stage I-II: 61% III-IV: 39%	G3: 100%	39	100	59.8± 6.4	Stage I-II: 59.0% III-IV: 41.0%	G3: 100%	TMA-IHC (NS)	0	5.0
**Numata, 2013 (Japan)**	Pancreatic cancer	No	Proteins: BRM, BRG1, BAF180, BAF47	34	61.8%	>64: 61.8%	Stage 0-III: 29.4% IV: 70.6%	G1: 41.2% G2-G3: 58.8%	34	44.1%	>64: 50%	Stage 0-III: 38.2% IV: 61.8%	G1: 18/34. 52.9% G2-G3: 47.1%	TMA-IHC (1)	0	5.0

(*Continued*)

						ARID1A−					ARID1A+					
**Park, 2015 (Korea)**	Clear cell renal cell caricnoma	No	No	213	26.3%	58.4± 10.4	Stage I-II: 71.7% III/IV: 28.3%	G1-G2: 43.7% G3-G4: 56.3%	77	23.4%	>55: 46.7%	Stage I-II: 85.7% III/ IV: 14.3%	G1-G2: 71.1% G3-G4: 29.9%	TMA-IHC (1)	2	6.9
**Rahman, 2013 (Japan)**	Endometrial cancer	No	p53, hMLH1, Her2, PTEN, ER, PR	27	100%	>59: 48.1%	FIGO Stage I-II: 63.0% III/IV: 37.0%	G1-G2: 85.2% G3: 14.8%	84	100%	59.8± 12.6	FIGO Stage I-II: 72.6% III/ IV: 27.4%	G1-G2: 30.2% G3: 29.8%	Whole-section IHC (1)	0	4.3
**Wang, 2011 (USA)**	Gastric cancer	NA	TP53	38	NA	NA	NA	NA	71	NA	NA	NA	NA	Exome-sequencing, IHC (NS)	3	5.0
**Wang, 2012 (China)**	Gastric cancer	No	No	115	27.8%	>54: 49.6%	NA	G1-G2: 13.1% G3: 86.9%	109	39.4%	>54: 57.8%	NA	G1-G2: 30.3% G3: 69.7%	Whole-section IHC (3)	5	5.0
**Wei. 2014 (China)**	Colorectal cancer	No	No	54	22.2%	>55 44.4%	Stage I-II: 31.5% III-IV: 68.5%	G1-G2: 74.1% G3: 25.9%	155	13.5%	>5551%	Stage I-II: 42.6% III-IV: 57.4%	G1-G2: 85.8% G3: 14.2%	Whole-section IHC (1)	2	5.0
**Wiegand, 2014 (Canada, Toronto cohort)**	Gastric carcinoma	Totally in 2 cohorts 102 cases (no data at follow-up; no clinicopatho-logical data)	MMR, HER2, EBV, p53	16	37.5%	71.6± 13.2	Stage I-II: 31.5%. III-IV: 68.5%	G2: 4. 25.0% G3: 12. 75.0%	64	31.3%	66.6± 11.1	Stage I-II: 34.3%. III-IV: 65.7%	G1-G2: 46.9% G3:. 53.1%	TMA-IHC (2)	10	2.0
**Wiegand, 2014 (Canada, Vancouver cohort)**	Gastric carcinoma	Totally in 2 cohorts 102 cases (no data at follow-up; no clinicopatho-logical data)	MMR, HER2, EBV, p53	29	25.6%	66.4± 12.2	Stage I-II: 44.1%. III-IV: 55.9%	G1-G2: 51.2% G3: 48.8%	134	27.6%	64.1± 10.5	Stage I-II: 45.5%. III-IV: 54.5%	G1-G2: 47.8% G3: 52.2%	TMA-IHC (2)	10	2.0

(*Continued*)

				ARID1A−					ARID1A+						
**Yan, 2014 (China)**	Gastric cancer	7 cases (follow-up lost)	E-cadherin	52	51.2%	54.4± 8.7	Stage I-II: 18.2% III-IV: 81.8%	G1-G2: 36.4% G3: 63.6%	137	32.3%	58.4± 10.5	Stage I-II: 16.3%. III-IV: 83.7%	G1-G2: 34.9% G3:. 65.1%	TMA-IHC (4)	4	5.0
**Ye, 2014 (USA)**	Colorectal carcinoma	45 TMA cores excluded for tissue loss	MMR	22	54.5%	Mean: 62.0 (range: 36-89)	Stage I-II: 45.4% III-IV: 54.6%	Low: 50%. High: 50%	235	48.1%	Mean: 49.7 Range (18-86)	Stage I-II: 50.2% III/ IV: 49.8%	Low: 87.2%. High and medullary: 12.8%	TMA-IHC (4)	0	4.8
**Yokoyama, 2014 (Japan)***	Ovarian Carcinoma	No	No	11	100%	52.0± 9.5	Stage III-IV: 100%	NA	35	100%	56±7.8	Stage III-IV: 100%	NA	Whole-section IHC (1)	5	5.3
**Zhang, 2012 (China)**	Breast cancer	No	HER2, p53, ER, PR, Ki67	22	100%	59.9± 11.3	Stage I-II: 90.9% III: 9.1%	NA	18	100%	60.3± 12.3	Stage I-II: 77.8% III: 22.2%	NA	mRNA real-time PCR (1)	4	5.4
**Zhang, 2014 (China)**	Breast cancer	No	HER2, p53, ER, PR, Ki67	63	100%	59.9 ± 9.9	Stage I-II: 93.7% III: 6.3%	NA	49	100%	61.3± 10.3	Stage I-II: 91.8% III: 8.2%	NA	Whole-section IHC (1)	0	3.1
**Zhao, 2014 (China)**	Breast cancer	No	p53, HER2, ER, PR	324	NA	>45: 66.7%	Stage I-II: 74.7% III: 25.3%	G1-G2: 71.9% G3: 28.1%	172	NA	>45: 53.5%	Stage I-II:, 89.5% III: 10.5%	G1-G2: 78.5% G3: 21.5%	TMA-IHC (4)	9	5.8

(*Continued*)

						ARIDIA−					ARID1A+				
**Total Studies (means, SDs and percent-ages are weighted with n values)**	**Gyneco-logical: 13 studies; Gastro-intestinal: 12 studies; Urological: 3 studies.**		**No other genes: 14 studies; p53: 8 studies; Her-2: 6 studies; MMR: 5 studies; Ki-67: 4 studies; Other: 6 studies.**	**1,701**	**54.9%**	**62.1± 12.6**	**TNM: (19 studies): I-II: 52.1%; III-IV: 47.9%; FIGO (3 studies): I-II: 66.1%; III-IV: 39.1%; NA: 3 studies**	**G1-G2: (17 studies) 47.0% G3: 53.0%; NA: 18 studies**	**3,950**	**52.7%**	**58.8± 11.2**	**TNM: (19 studies): I-II: 63.6%%; III-IV: 36.4%; FIGO (3 studies): I-II: 83.0%; III-IV: 17.0%; NA (3 studies)**	**G1-G2: (17 studies) 51.3% G3: 48.7%; NA: 18 studies**	**TMA-IHC:17 studies; Whole section IHC: 8 studies; NGS: 1 study; mRNA real-time PCR: 1 study; IHC+exome sequencing:1 study**	**3±3 (range: 0-10)**	**4.7±1.8 years**

Notes:

If not specified (i.e. FIGO staging system), the used staging system is TNM.

*Here the data are calculated on the basis of only 46 patients, all in III-IV Stage, because survival data are calculated only in this patients' subgroup.

Abbreviations: FIGO: International Federation of Gynecology and Obstetrics ; IHC: immunohistochemistry; NA: not available; NGS: next-generation sequencing; PCR: polymerase chain reaction; TMA-IHC: tissue micro-array immunohistochemistry; TNM: tumor, nodes, metastasis; NS: not specified.

IHC: numbers are used to indicate the specific antibody used in the study for immunohistochemical analysis (if applicable):

1: mouse monoclonal antibody (Cat.# sc-32761, Santa Cruz Biotechnology, TX)

2: rabbit polyclonal antibody (ARID1A; HPA005456, Sigma-Aldrich St Louis, MO)

3: mouse monoclonal antibody (clone 3H2, Abgent Inc., San Diego, CA)

4: mouse monoclonal anti-ARID1A antibody, clone 3H2 (Sigma-Aldrich)

Supplementary Table S2: Methodological Quality Of Cohort Studies Included In The Meta-Analysis*

First author, publication year	Represen-tativenessof the exposed cohort	Selection of the unex-posedcohort	Ascertain-mentof exposure†	Outcome of interestnot presentat start of study††	Control forimportant factor or additional factor†††	Assessment of outcome	Follow-uplong enough for outcomesto occur††††	Adequacy offollow-upof cohorts	Total quality scores
Cho, 2013	*	*	*	*	*	*	-	*	7
Chou, 2014	*	*	*	-	**	*	*	*	8
Fadare, 2012	*	*	*	*	-	*	-	*	6
Faraj, 2014	*	*	*	*	*	*	*	-	7
Katagiri, 2012 (cervical)	*	*	-	*	**	*	-	*	7
Katagiri, 2012 (ovarian)	*	*	-	*	**	*	-	*	7
Kim, 2014	*	*	*	-	**	*	-	*	7
Lee, 2014	*	*	*	-	*	*	*	*	7
Lichner, 2013	*	*	*	-	-	*	-	*	7
Lowery, 2012	*	*	*	-	*	*	*	*	7
Maeda, 2010	*	*	*	-	*	*	-	*	6
Nelson, 2013	*	*	*	-	**	*	-	-	6
Numata, 2013	*	*	-	-	**	*	-	*	6
Park, 2015	*	*	*	*	*	*	*	*	8
Rahman, 2013	*	*	*	*	**	*	-	*	8
Wang, 2011	*	*	*	-	*	*	-	*	6
Wang, 2012	*	*	*	-	*	*	*	*	7
Wei, 2014	*	*	*	-	*	*	*	*	7
Wiegand, 2014	*	*	*	*	**	*	*	*	9
Yan, 2014	*	*	*	-	*	*	-	-	5
Ye, 2014	*	*	*	-	*	*	*	*	7
Yokoyama, 2014	*	*	*	*	*	*	*	*	8
Zhao, 2014	*	*	*	-	**	*	*	*	8

Original studies were analyzed in the quality assessment.

*A study could be awarded a maximum of one star for each item except for the item Control for important factor or additional factor. The definition/explanation of each column of the Newcastle-Ottawa Scale is available at http://www.ohri.ca/programs/clinical_epidemiology/oxford.htm.

†For this index, one star was given if in Method section the ARID1A assessment was calculated with genetic analysis, whole section-IHC, TMA-IHC with at a least 2 cores per case or with an adjunctive method or technique as control.

††Being outcome of interest mortality, we took as outcome of interest for assessment of quality if the disease-specific survival or the recurrence rate was assessed.

†††A maximum of 2 stars could be awarded for this item. Studies that controlled their survival analyses for at least two confounders received one star, whereas studies that controlled genetic status of other gene/genes, an additional star.

††††A cohort study with a mean/median follow-up time $\geq$ 5 y (60 months) takes one star.

Supplementary Table S3: Results Of Univariable Moderator Analyses For All-Cause Mortality, Recurrence Of Disease And Adjusted HR Estimates For All-Cause Mortality And Recurrence By ARID1A Status

Moderator Variable		All-cause mortality	Recurrence	Adjusted HR estimates for all-cause mortality	Adjusted HR estimates for recurrence
**Study characteristics**	Studies	26	7	19	10
	Patients: ARID1A+ vs. ARID1A-	3,735 vs. 1,633	373 vs. 454	3,139 vs. 1,062	871 vs. 497
**System (0 = urological system [ref.] vs.1 = gastrointestinal system vs. 2 = gynaecological system)**	*p*-value	0.07 (gastro) 0.43 (gyneco)	0.11 (gyneco)	0.80 (gastro) 0.53 (gyneco)	0.51 (gastro) 0.68 (gyneco)
	Slope	0.36 (gastro) 0.15 (gyneco)	-0.19 (gyneco)	0.14 (gastro) 0.35 (gyneco)	-0.38 (gastro)0.24 (gyneco)
	Studies	26	7	19	10
**Sample size**	*p*-value	0.44	0.58	0.27	0.86
	Slope	-0.0002	0.0003	-0.0006	-0.0004
	Studies	26	7	19	10
**Age difference**	*p*-value	0.96	0.84	0.72	0.41
	Slope	0.00014	0.0139	0.0229	0.078
	Studies	16	6	14	8
**% Females difference**	*p*-value	0.38	0.76	0.66	0.52
	Slope	0.0088	-0.0041	0.0120	-0.03
	Studies	25	7	17	9
**TNM 1–2 difference**	*p*-value	0.62	0.11	0.96	0.09
	Slope	0.0024	0.00167	-0.0008	0.03
	Studies	20	6	15	8
**TNM 3–4 difference**	*p*-value	0.31	0.36	0.75	0.24
	Slope	0.0039	-0.0036	0.0046	-0.017
	Studies	21	6	16	8
**Grade 1–2 difference**	*p*-value	**0.01**	0.80	0.20	0.78
	Slope	**0.0077**	0.0007	0.0149	0.0025
	Studies	**16**	5	13	7
**Grade 3–4 difference**	*p*-value	0.19	0.39	0.30	0.43
	Slope	0.0082	-0.0037	0.0157	-0.0179
	Studies	15	5	12	7

(*Continued*)

Moderator Variable		All-cause mortality	Recurrence	Adjusted HR estimates for all-cause mortality	Adjusted HR estimates for recurrence
**Newcastle-Ottawa scale**	*p*-value	0.46	0.36	0.90	0.33
	Slope	-0.06	-0.14	-0.02	-0.23
	Studies	26	7	19	10
**Number of adjustments**	*p*-value	0.83	**0.003**	0.29	0.86
	Slope	-0.0055	**0.11**	0.056	-0.00115
	Studies	26	**7**	19	10
**Method of ARID1A analysis (0 = TMA-IHC [ref.] vs. 1 = other methods)**	*p*-value	0.69	0.09	0.56	0.66
	Slope	0.20	-0.23	-0.24	0.22
	Studies	26	7	19	10
**Country (0 = Asia [ref.] vs. 1 = other)**	*p*-value	**0.04**	0.82	0.35	0.36
	Slope	**0.27**	0.04	-0.33	-0.41
	Studies	**26**	7	19	10
**Mean follow-up period**	*p*-value	0.84	0.79	0.30	0.11
	Slope	-0.05	0.11	-0.17	0.21
	Studies	26	7	19	10

Supplementary Table S4: Results Of Multivariable Meta-Regression Analyses Of Moderators Variables

Significant Multivariable Moderators	All-cause mortality	Recurrence	Adjusted HR estimates for all-cause mortality	Adjusted HR estimates for recurrence
**Studies**<br>**N**	15<br>4,573	5<br>946	No multivariate analysis was made since in the univariate analysis no moderator reached a $p < 0.20$	8<br>1,300
**Moderator 1**	Grade 1–2 difference: Mean difference between groups = 1.17<br>z = 3.00<br>$p = 0.0027$	Covariates:<br>Mean: 2.8<br>z = 1.68<br>$p = 0.09$	-	TNM 1–2 difference:Mean difference between groups = 10.59<br>z = 1.65<br>$p = 0.09$
**Moderator 2**	Gastrointestinal system (38%):<br>z = 1.47<br>$p = 0.14$<br>Gynecological System (46%):<br>z=-0.82<br>$p = 0.41$<br>Gastrointestinal + gynecological systems vs. urological:<br>$p = 0.02$	Gynecological System (57%):<br>z = 0.10<br>$p = 0.61$	-	-
**Moderator 3**	-	TNM 1–2 difference:<br>Mean: 11.7<br>z = -0.01 $p = 0.99$	-	-
**Overall Model**	Q = 35.99<br>$p < 0.0001$<br>$R^2 = 0.76$	Q = 10.44<br>$p = 0.06$<br>$R^2 = 0.92$	-	Q = 26.49<br>$p = 0.0004$<br>$R^2 = 0.41$

Supplementary Table S5: All-Cause Mortality And Recurrence Risk For Strata Of Different Moderators

			Outcome			
**Moderator**	**Strata**	**Analysis details**	**All-causemortality***	**Recurrence***	**Adjusted HR estimates for All-cause mortality**	**Adjusted HR estimates for recurrence**
**System**	**Gastrointestinal**<br>**Gynecological**<br>**Urological**	***Pooled estimate***,<br>HR (95%CI)<br>*p*-value for HR<br>***Heterogeneity***, I^2<br>(*p*-value)<br>***Number of studies***<br>***Number of***<br>***Subjects***<br>***Pooled estimate***,<br>HR (95%CI)<br>*p*-value for HR<br>***Heterogeneity***, I^2<br>(*p*-value)<br>***Number of studies***<br>***Number of***<br>***Subjects***<br>*P*-value[a]<br>***Pooled estimate***,<br>HR (95%CI)<br>*p*-value for HR<br>***Heterogeneity***, I^2<br>(*p*-value)<br>***Number of studies***<br>***Number of***<br>***Subjects***<br>***p*-value** [a]	1.22 (0.89–1.68)<br>0.22<br>84 (<0.0001)<br>10<br>1,577<br>0.99 (0.90–1.10)<br>0.95<br>64 (0.001)<br>12<br>2,748<br>0.84 (0.47–1.50)<br>0.56<br>96 (<0.0001)<br>4<br>963<br>0.41	-<br>-<br>-<br>0<br>0<br>1.06 (0.74–<br>1.52)<br>0.75<br>53 (0.09)<br>4<br>262<br>**1.15 (1.07–**<br>**1.23)**<br>**< 0.0001**<br>0 (0.46)<br>3<br>841<br>0.67	1.10 (0.67–1.79)<br>0.71<br>74 (<0.0001)<br>8<br>1,176<br>1.34 (0.84–2.14)<br>0.22<br>49 (0.05)<br>8<br>2342<br>0.91 (0.19–4.23)<br>0.91<br>81 (0.005)<br>3<br>673<br>0.79	1.69 (0.59–4.83)<br>0.33<br>86 (0.001)<br>3<br>265<br>1.78 (0.72–4.43)<br>0.21<br>70 (0.02)<br>4<br>262<br>**2.25 (1.60–3.15)**<br>**< 0.0001**<br>78 (<0.0001)<br>3<br>841<br>0.81
**Country**	**Asia**<br>Other	***Pooled estimate***,<br>HR (95%CI)<br>*p*-value for HR<br>***Heterogeneity***, I^2<br>(*p*-value)<br>***Number of studies***<br>***Number of***<br>***Subjects***<br>***Pooled estimate***,<br>HR (95%CI)<br>*p*-value for HR<br>***Heterogeneity***, I^2<br>(*p*-value)<br>***Number of studies***<br>***Number of***<br>***Subjects***<br>***p*-value** [a]	1.12 (0.99–1.27)<br>0.07<br>86 (<0.0001)<br>16<br>2,506<br>0.87 (0.67–1.14)<br>0.31<br>79 (<0.0001)<br>10<br>2,782<br>0.09	1.11 (0.92–<br>1.34)<br>0.29<br>57 (0.03)<br>6<br>404<br>**1.16 (1.04–**<br>**1.28)**<br>**0.005**<br>-<br>1<br>699<br>0.69	0.94 (0.50–1.79)<br>0.86<br>71 (0.005)<br>13<br>1,912<br>1.30 (0.90–1.88)<br>0.16<br>60 (0.003)<br>6<br>2,27<br>90.39	2.37 (1.54–3.66)<br><0.0001<br>88(<0.0001)<br>7<br>721<br>1.52 (0.66–3.46)<br>0.32<br>41 (0.12)<br>3<br>647<br>0.35

(Continued)

			Outcome			
**Moderator**	**Strata**	**Analysis details**	**All-causemortality***	**Recurrence***	**Adjusted HR estimates for All-cause mortality**	**Adjusted HR estimates for recurrence**
**Study quality[b]**	**NOS score < 7**<br>**NOS score ≥ 7**	***Pooled estimate***, HR (95%CI)<br>*p*-value for HR<br>***Heterogeneity***, I^2<br>(*p*-value)<br>***Number of studies***<br>***Number of Subjects***<br>***Pooled estimate***, HR (95%CI)<br>*p*-value for HR<br>***Heterogeneity***, I^2<br>(*p*-value)<br>***Number of studies***<br>***Number of Subjects***<br>***p*-value [a]**	1.06 (0.84–1.34)<br>0.61<br>89 (<0.0001)<br>15<br>2,083<br>0.99 (0.85–1.15)<br>0.87<br>81 (<0.0001)<br>11<br>3,205<br>0.61	All studies had a NOS score ≥ 7	1.44 (0.99–2.09)<br>0.06<br>0 (0.46)<br>3<br>235<br>1.13 (0.76–1.69)<br>0.54<br>71 (<0.0001)<br>16<br>3,956<br>0.40	**3.09 (1.13–8.48)**<br>**0.03**<br>0 (1.00)<br>1<br>22<br>**1.84 (1.13–3.00)**<br>**0.01**<br>78 (<0.0001)<br>9<br>1,346<br>0.37

*For these outcomes, RRs instead of HRs were used.
Bolded RR or HR values: $p < 0.05$
Abbreviations: NOS, Newcastle-Ottawa Scale
[a]The *p*-value for the *t*-test between the two statistical analysis strata according to meta-regression procedure.
[b]Stratification was performed by median NOS score and number of adjustments as appropriate.
